# A Comparison of Anthropometric, Metabolic, and Reproductive Characteristics of Young Adult Women from Opposite-Sex and Same-Sex Twin Pairs

**DOI:** 10.3389/fendo.2014.00028

**Published:** 2014-03-07

**Authors:** Pirkko Korsoff, Leonie H. Bogl, Päivi Korhonen, Antti J. Kangas, Pasi Soininen, Mika Ala-Korpela, Richard J. Rose, Risto Kaaja, Jaakko Kaprio

**Affiliations:** ^1^Satakunta Central Hospital, Pori, Finland; ^2^Department of Public Health, University of Helsinki, Helsinki, Finland; ^3^Satakunta Central Hospital, Pori and University of Turku, Turku, Finland; ^4^Computational Medicine, Institute of Health Sciences, Faculty of Medicine, University of Oulu, Oulu, Finland; ^5^NMR Metabolomics Laboratory, School of Pharmacy, University of Eastern Finland, Kuopio, Finland; ^6^Oulu University Hospital, Oulu, Finland; ^7^Computational Medicine, Medical Research Council Integrative Epidemiology Unit, School of Social and Community Medicine, University of Bristol, Bristol, UK; ^8^Department of Psychological and Brain Sciences, Indiana University, Bloomington, IN, USA; ^9^Department of Mental Health and Substance Abuse Services, National Institute for Health and Welfare, Helsinki, Finland; ^10^Institute for Molecular Medicine FIMM, Helsinki, Finland

**Keywords:** prenatal androgen exposure, twin testosterone transfer hypothesis, opposite-sex twin pairs, anthropometrics, reproductive history, lipoprotein profile

## Abstract

**Background:** Prenatal exposure to androgens has been linked to masculinization of several traits. We aimed to determine whether putative female intra-uterine exposure to androgens influences anthropometric, metabolic, and reproductive parameters using a twin design.

**Methods:** Two cohorts of Finnish twins born in 1975–1979 and 1983–1987 formed the basis for the longitudinal FinnTwin16 (FT16) and FinnTwin12 (FT12) studies. Self-reported anthropometric characteristics, disease status, and reproductive history were compared between 679 same-sex (SS) and 789 opposite-sex (OS) female twins (mean age ± SD: 34 ± 1.1) from the wave 5 of data collection in FT16. Serum lipid and lipoprotein subclass concentrations measured by nuclear magnetic resonance spectroscopy were compared in 226 SS and 169 OS female twins (mean age ± SD: 24 ± 2.1) from the wave 4 of data collection in FT12 and FT16.

**Results:** Anthropometric measures, the prevalence of hypertension and diabetes mellitus type 2 did not differ significantly between females from SS and OS twin pairs at age 34. Similarly, the prevalence of infertility, age at first pregnancy and number of induced and spontaneous abortions did not differ significantly between these two groups of women. The serum lipid and lipoprotein profile did not differ between females from SS and OS twins at age 24.

**Conclusion:** We found no evidence that androgen overexposure of the female fetus affects obesity, metabolic profile, or reproductive health in young adult females. However, these results do not exclude the possibility that prenatal androgen exposure in females could be adversely associated with these phenotypes later in life.

## Introduction

Hyperandrogenism and insulin resistance are key features of polycystic ovary syndrome (PCOS), and women with PCOS are consequently at an increased risk of developing type 2 diabetes mellitus and the metabolic syndrome (1). Increased ovarian androgen production leads to premature adrenarche, menstrual irregularity, acne, hirsutism, and infertility by means of elevated luteinizing hormone to follicle stimulating hormone production and hyperinsulinemia (2, 3). In addition to these important reproductive outcomes, hyperandrogenism is associated with an adverse metabolic profile, including obesity, particularly abdominal obesity (4), hypertension (5), insulin resistance (6), type 2 diabetes (7), dyslipidemia (8, 9), and subclinical atherosclerosis leading to increased cardiovascular morbidity (10, 11). The onset and duration of exposure to excess androgens required to induce metabolic and reproductive anomalies is unknown. However, it has been hypothesized that intra-uterine exposure to androgens can accelerate hyperandrogenism-related phenotypes through epigenetic mechanisms (12, 13). A natural experiment to test the intra-uterine exposure hypothesis is the existence of same-sex (SS) and opposite-sex (OS) twin pairs, with the premise that females in OS twin pregnancies may be exposed to androgens from the male fetus (14), while females from female–female pairs are not. In a Swedish study of over 17,000 female twins, females from OS twin pairs showed a more adverse anthropometric and metabolic profile than females from SS twin pairs. However, the differences between the zygosity groups were only observed in those females who were over 60 years of age (15).

We examined anthropometric and metabolic signs of hyperandrogenism and reproductive health in two large longitudinal studies of twins, which include females from SS and OS DZ twin pairs. The analysis is restricted to DZ pairs to avoid confounding from the greater variability in placentation patterns among monozygotic twin pairs. Variables related to anthropometric characteristics, disease status, and reproductive history were examined in the wave 5 study of the Finntwin16 (FT16) study, when the subjects were aged 34 years on average, while information on lipids was available from two clinical subsamples studied in their 20s. We found no evidence for any phenotype for differences between women from SS and OS twin pairs.

## Materials and Methods

### The twin cohorts

The sample was derived from two population-based longitudinal studies, FinnTwin16 and FinnTwin12 (FT12) (16, 17). Both are longitudinal studies of behavioral development and health habits of Finnish twins initially enrolled during adolescence, and repeatedly assessed by self-report questionnaires. FT12 included five consecutive birth cohorts of Finnish twins born in 1983–1987. Questionnaires were mailed to twin individuals in the autumn of the year in which their birth cohort reached age 11 (90% of the responses were received by the end of that year), and subsequent follow-up assessments were made at 14, 17.5, and ~22 years. A subsample of the wave four participants was assessed in person in Helsinki, at which time a fasting blood sample was taken. In FT16, the baseline survey questionnaire was sent to all Finnish twins born in 1975–1979 within 2 months after their 16th birthday (response rate of 88%) and individuals were mailed four follow-up questionnaires at 17, 18.5, ~25, and 34 years. After the fourth wave, some of the twins were invited to more detailed clinical assessments in Helsinki, and a blood sample was taken in the morning of the assessment. Serum lipid and lipoprotein measurements were derived from these two sets of blood samples collected when the twins were in their mid-20s. The fifth wave of data collection of the FT16 cohort was done between October 2010 and November 2011 for Finnish speaking subjects and in the spring of 2012 for Swedish speaking subjects. The invitation to take part in an internet survey was sent to all twins in the cohort (born 1975–1979) living in Finland irrespective of earlier participation. Of the 6132 twins that we contacted, 4246 provided adequate data, a response rate of 69%. There were 679 female twins of SS pairs and 789 female twins from OS pairs. Mean age was 34.0 years, SD 1.13, range 31.9–37.3, with no difference by zygosity status.

Subjects completed questionnaires on anthropometric characteristics, medical status, and reproductive history. BMI (kilograms per square meter) was calculated from self-reported height (meters) and weight (kilograms). Waist circumference was self-measured and self-reported at the level midway between the lowest rib and the iliac crest according to the instructions supplied with a picture. Medical history was assessed with the question: “Has a doctor ever told you that you suffer or have suffered from hypertension, diabetes mellitus type 1 or type 2?” Response options were “yes” or “no” for each item. Reproductive history was assessed as follows: are you currently pregnant (yes/no), do you have children of which you are the biological parent (yes/no), have you ever had a spontaneous abortion (yes/no) and the number of children (open answer). The age at first pregnancy was computed from the mother birth year and year of first birth. Infertility was measured by one item: “have you ever tried to become pregnant for more than 1 year without achieving a pregnancy?”

### Sample characteristics of the twins with lipid measurements

Serum lipid and lipoprotein subclass concentrations were measured by proton nuclear magnetic resonance (NMR) spectroscopy (18, 19). The 14 lipoprotein subclass sizes determined by this methodology are as follows: chylomicrons and extremely large VLDL particles (with particle diameters from ~75 nm upwards), five different VLDL subclasses, namely, very large VLDL (average particle diameter of 64.0 nm), large VLDL (53.6 nm), medium VLDL (44.5 nm), small VLDL (36.8 nm), very small VLDL (31.3 nm); IDL (28.6 nm), three LDL subclasses as large LDL (25.5 nm), medium LDL (23.0 nm), and small LDL (18.7); and four HDL subclasses as very large HDL (14.3 nm), large HDL (12.1 nm), medium HDL (10.9 nm), and small HDL (8.7 nm). We grouped extremely large, very large, and large VLDL to “large VLDL,” small and very small VLDL to “small VLDL,” IDL and large LDL to “large LDL” and very large and large HDL to “large HDL.” Thus, three subclasses (large, medium, and small) of VLDL, LDL, and HDL were analyzed. The mean particle size for VLDL, LDL, and HDL particles was calculated by weighting the corresponding subclass diameters with their particle concentrations. Apolipoprotein B (apoB) and apolipoprotein A-1 (apoA-1) were estimated from an extended version of the Friedewald formula (20). NMR spectroscopy measurements were available for 436 DZ female twins (249 SS and 187 OS twins). The exclusion criteria were lipid-lowering medication (*n* = 1) and pregnancy (*n* = 40). Thus, the final sample comprised 395 DZ female twins (226 SS and 169 OS twins). Mean age was 23.9 years, SD 2.1, range 21–29, with no difference by zygosity status. Data collection and analysis were approved by the ethics committee of the Department of Public Health of the University of Helsinki, ethics committee of the Helsinki University Hospital District, and the Institutional Review Board (IRB) of Indiana University. Written informed consent was obtained from all participants.

### Statistical methods

For the wave 5 questionnaire data of FT16, we tested differences between women from SS and OS pairs by an adjusted Wald test for continuous variables and Chi^2^/design-based F for categorized variables to take into account the clustering of twins in twin pairs. Sample sizes varied somewhat due to non-response to selected items.

For the wave 4 lipoprotein data of FT12 and FT16 subjects, differences between SS and OS DZ female twins were tested by the Wald tests for independent samples (*t-*tests adapted for clustered twin data). Because the distribution of most lipid measures was highly skewed, variables were standardized by cohort and transformed using rank transformation methods in R version 2.14.0. The standard errors were corrected for clustering of twin pairs by survey methods (21). Principal component analysis was used to determine the number of principal components that explain most of the variance of the studied lipids and lipoproteins. As the strong correlations among these metabolites makes the traditional Bonferroni correction for multiple testing too conservative, the number of principal components provides a more permissive *P*-value threshold. In this study, the first five principal components explained more than 95% of the variance, allowing associations to be significant at *P* < 0.01 after the Bonferroni correction. Sample sizes vary slightly in statistical analyses because of missing data (*n* = 353–395). Statistical analyses were conducted using the Stata statistical software package (release 12.0; Stata Corporation, College Station, TX, USA).

## Results

Characteristics for females from SS and OS twin pairs are shown in Table 1. The females were normal weight based on BMI. There were no significant differences in height or adiposity measures (BMI and waist circumference) between females from SS and OS twin pairs. The prevalence of hypertension, type 1 diabetes mellitus and type 2 diabetes mellitus was low in both zygosity types and did not differ significantly between SS and OS female twins.

**Table 1:** **Obesity measures, disease status, and reproductive characteristics for females from same-sex (SS) and opposite-sex (OS) dizygotic twin pairs**.

Variable	SS females (*n* = 679)	OS females (*n* = 789)	*P*-value
Height (m), mean and SD	1.66 ± 6.1	1.66 ± 5.6	0.19
BMI (kg/m^2^), mean and SD	24.2 ± 4.9	23.9 ± 4.4	0.34
Waist circumference (cm) mean and SD	81.9 ± 12.3	81.4 ± 11.7	0.48
Hypertension (%)	1.78	1.81	0.97
Type 1 diabetes (%)	0.89	0.65	0.60
Type 2 diabetes (%)	0.75	1.17	0.42
Current pregnancy (%)	10.2	7.4	0.06
Age at the first pregnancy, years	29.6	29.2	0.53
Prevalence of biological children (%)	62.2	66.1	0.12
Number of biological children (%)
1 Child	32.1	31.3	
2 Children	44.5	46.4	
3 Children	16.4	16.2	
4 Children	4.5	3.8	
5 Children	2.6	2.3	0.97
Spontaneous abortions (%)
Once	16.2	13.6	
2 Or more	4.1	4.2	0.37
Infertility of >1 year duration (%)	15.8	15.5	0.93

**P*-value from the Wald test for continuous variables and Chi^2^/design-based F for categorized variables. Mean values (±SD) or percentages*.

*Missing values for height (*n* = 10), BMI (12), and waist (*n* = 61)*.

*Wave 5 of FinnTwin16*.

The reproductive history was similar between the zygosity groups of females. There were no differences in the age at the first pregnancy, the number of biological children, and the number of spontaneous abortions between females from SS and OS twin pairs. Females from SS pairs were more likely to be currently pregnant (10.2 vs. 7.4%, *P* = 0.058). The prevalence of infertility of more than 1 year duration did not differ significantly between the two groups of women (Table 1).

Serum lipids and lipoproteins concentrations for females from SS and OS twin pairs are shown in Table 2. Females from SS twin pairs had higher concentrations of serum triglycerides (mean ± SD: 1.13 ± 0.51 vs. 1.01 ± 0.43, *P* = 0.038). However, only a *P*-value below 0.01 was considered statistically significant after Bonferroni correction. None of the other serum lipid and lipoprotein subclass concentrations differed significantly between females from SS and OS twin pairs (Table 2).

**Table 2:** **Serum lipid and lipoprotein profile for females from same-sex (SS) and opposite-sex (OS) twin pairs**.

	SS females (*n* = 226)	OS females (*n* = 169)	*P*-value
**LIPOPROTEIN PARTICLE CONCENTRATIONS**			
Large VLDL (and chylomicrons) (nmol/l)	4.18 ± 4.01	3.51 ± 3.12	0.14
Medium VLDL (nmol/l)	13.79 ± 8.63	12.28 ± 6.70	0.17
Small VLDL (nmol/l)	54.08 ± 17.59	50.07 ± 16.79	0.05
Large LDL (nmol/l)	241.35 ± 56.90	230.61 ± 58.50	0.07
Medium LDL (nmol/l)	119.18 ± 30.70	114.03 ± 31.03	0.12
Small LDL (nmol/l)	136.64 ± 33.97	130.33 ± 33.99	0.10
Large HDL (μmol/l)	2.05 ± 0.78	2.06 ± 0.68	0.92
Medium HDL (μmol/l)	2.46 ± 0.45	2.37 ± 0.46	0.07
Small HDL (μmol/l)	4.87 ± 0.54	4.76 ± 0.56	0.06
**LIPOPROTEIN PARTICLE SIZE**			
VLDL diameter (nm)	36.53 ± 1.48	36.39 ± 1.47	0.50
LDL diameter (nm)	23.60 ± 0.18	23.60 ± 0.16	0.86
HDL diameter (nm)	10.10 ± 0.25	10.12 ± 0.23	0.45
**APOLIPOPROTEINS**			
ApoA-1 (g/l)	1.89 ± 0.22	1.88 ± 0.22	0.63
ApoB (g/l)	0.84 ± 0.19	0.81 ± 0.17	0.31
ApoB/ApoA-1 ratio	0.45 ± 0.10	0.44 ± 0.09	0.57
**TRIGLYCERIDES**			
Total triglycerides (mmol/l)	1.13 ± 0.51	1.01 ± 0.43	0.04
Extremely large VLDL-TG (mmol/l)	0.01 ± 0.01	0.01 ± 0.01	0.05
Total VLDL-TG (mmol/l)	0.70 ± 0.44	0.62 ± 0.35	0.13
**CHOLESTEROL**			
Total cholesterol (mmol/l)	4.99 ± 0.90	4.86 ± 0.89	0.23
IDL-C (mmol/l)	0.70 ± 0.16	0.68 ± 0.16	0.38
LDL-C (mmol/l)	1.72 ± 0.48	1.66 ± 0.49	0.20
HDL-C (mmol/l)	2.01 ± 0.42	1.99 ± 0.37	0.67
HDL2-C (mmol/l)	1.49 ± 0.45	1.47 ± 0.39	0.84
HDL3-C (mmol/l)	0.53 ± 0.06	0.52 ± 0.05	0.63
HDL-C/LDL-C ratio	1.26 ± 0.47	1.30 ± 0.44	0.43

**P*-value from the Wald tests for independent samples. Mean values (±SD)*.

*Statistical significance at *P* < 0.01 after Bonferroni correction*.

*Wave 4 clinical subsample data from FinnTwin12 and FinnTwin16*.

## Discussion

The hypothesis that prenatal hormone transfer from the male co-twin may result in masculinization of females, and therefore predispose them to endocrine disorders can be tested by comparing females from SS and OS twin pairs, i.e., females *in utero* with a male as compared to a female co-twin. Therefore, if prenatal androgen exposure influences phenotypes related to hyperandrogenism, females from OS twin pairs are expected to have a higher BMI, higher prevalence of type 2 diabetes and hypertension and a more adverse lipid profile than females from SS pairs. However, in the present study we did not find significant differences in anthropometric measures, disease status, and reproductive history between females from SS and OS twin pairs. We cannot exclude the possibility of minor differences despite a fairly substantial sample size.

The concept that individual variability in sex-related traits may be influenced by variations in hormonal exposure during fetal development is interesting and comes from animal studies with placentation patterns, which are quite different from human twin pregnancies (14). Female fetuses developing between two males tend to show masculinized anatomical, physiological, and behavioral traits as adults. Female fetuses developing without adjacent males, on the other hand, tend to show more feminized traits as adults. These traits include permanently altered hormone levels, reproductive organs, aggressive behaviors, secondary sex ratios, and susceptibility to endocrine disruption. This intra-uterine effect is due to the transfer of testosterone from male fetuses to adjacent fetuses (14).

It is questionable whether prenatal testosterone transfer occurs in humans. Thus far direct evidence for the existence of prenatal testosterone transfer in females from OS twin pregnancies is missing. Testosterone is a steroid hormone; therefore it has the ability to diffuse through the amniotic fluid between fetuses (22). In addition, hormones can transfer among fetuses through the mother’s bloodstream (23). Evidence is mounting, however, for *in utero* testosterone excess, together with gestational hyperglycemia, contributing to either early differentiation of PCOS or phenotypic amplification of its genotypes. Abnormal endocrine, ovarian, and hyperinsulinemic traits are detectable as early as 2 months of age in daughters of women with PCOS, with adiposity enhancement of hyperinsulinemia during childhood potentially contributing to hyperandrogenism and luteinizing hormone excess by adolescence (12).

There is indirect evidence that human fetuses gestated with a male co-twin may be masculinized in development, perhaps due to the influence of prenatal androgens: the so-called twin testosterone transfer hypothesis. Results from studies in humans using a number of traits that show distinct sexual dimorphism are conflicting. Generally, the evidence for traits such as perception and cognition is more consistent than for behaviors (24). For example, we have shown a decreased prevalence of left-handedness and better mental rotation performance among females with male co-twins as compared to females with female co-twins (25, 26), a finding consistent with the intra-uterine exposure hypothesis. On the other hand, for a variety of personality and fertility traits, we have shown no differences between females from like and OS DZ pairs (27). Some studies have reported that females from OS twin pairs show an increased tooth size (28), adverse anthropometric, and metabolic parameters (15), increased alcohol use disorder symptoms (29), increased risk for alcohol dependence (30), greater sensation seeking (31), and enhanced aggressive behaviors (32) as compared to female twins from SS twin pairs. However, the present literature is far from consistent, and negative reports exist for several traits, including, anthropometric measures (33), birth weight (34), disordered eating (35, 36), and fertility (27).

The only twin study examining the prenatal exposure effects of androgens on the prevalence of PCOS found no differences between women from OS (480 women) and SS twin pairs (711 women) (37). PCOS was defined as less than nine natural menstrual cycles a year combined with either hirsutism or acne, which is not a fully satisfactory definition of PCOS. Our results were in good agreement with the study of Kuijper et al. (37), although we did not study PCOS *per se*, but compared parameters related to the androgenic phenotype including BMI, waist circumference, hypertension, type 2 diabetes mellitus, infertility, and the serum lipid profile.

According to the prenatal testosterone exposure hypothesis, the women with a twin brother in the present study would be expected to be more prone to the hyperandrogenism-related phenotypes. However, they tended to have a more favorable serum lipoprotein profile than women from SS pairs, albeit these differences did not reach statistical significance after correction for multiple comparisons. Testosterone administration has been shown to decrease measured HDL, which is associated with atherosclerosis (38). It is possible that the higher HDL concentrations associated with female gender contradicts the effect of intra-uterine hyperandrogenism.

An early onset of menarche has been associated with body fatness (39) and early menarche has been suggested to trigger the development of the metabolic syndrome and incidence of PCOS (40). We previously reported that the women from OS DZ twin pairs had a significantly higher mean age at menarche (13.3 years) compared to the women from DS pairs (13.1 years) (41). This is a small difference and one that is unlikely to be of clinical relevance.

Non-classical congenital adrenal hyperplasia (NCAH) due to 21-hydroxylase deficiency is the most common inherited disorder of adrenal steroid biosynthesis. Patients with the classic form of NCAH show androgen excess, with or without salt wasting. The factors that associate with increased risk for adverse metabolic consequences cluster in women with NCAH and include obesity, hypertension, and insulin resistance. The androgen excess may independently contribute to this increased risk due to atherogenic lipid profiles (42). Comparison of metabolic parameters in women with PCOS, women with NCAH, and healthy control women showed that metabolic parameters were comparable among women with NCAH, lean women with PCOS, and healthy control women whereas metabolic dysfunction was evident in the obese women with PCOS (43).

There are some weaknesses in our study. We measured only phenotypes related to hyperandrogenism and not the plasma levels of androgens. As for PCOS, we did not have any questionnaire items concerning oligomenorrea nor clinical signs for hyperandrogenism (e.g., hirsutism). The subjects of the present study were young adults and mostly healthy, which may explain why we did not observe differences in disease status between SS and OS female twins. Moreover, we cannot extrapolate our findings to middle-aged or older females. The strength of our study includes the twin study design, population-based sampling, and high response rates, as well as the comprehensive analysis of serum lipoprotein subclasses.

In conclusion, anthropometric characteristics, disease status, and reproductive history did not differ between females from SS and OS twin pairs. Thus, we found no evidence for the hypothesis that prenatal hormone transfer from the male co-twin may result in masculinization of females in regard to these PCOS-related phenotypes. However, these results do not exclude the possibility that prenatal androgen exposure in females could be associated with these phenotypes later in life.

## Conflict of Interest Statement

Antti J. Kangas, Pasi Soininen, and Mika Ala-Korpela are shareholders of BrainsTRL-79hake Ltd., a startup company offering NMR-based metabolite profiling. The other co-authors declare that the research was conducted in the absence of any commercial or financial relationships that could be construed as a potential conflict of interest.
